# Sulforaphane diminishes moonlighting of pyruvate kinase M2 and interleukin 1β expression in M1 (LPS) macrophages

**DOI:** 10.3389/fimmu.2022.935692

**Published:** 2022-08-02

**Authors:** Sheyda Bahiraii, Martin Brenner, Fangfang Yan, Wolfram Weckwerth, Elke H. Heiss

**Affiliations:** ^1^ Department of Pharmaceutical Sciences, University of Vienna, Vienna, Austria; ^2^ Vienna Doctoral School of Pharmaceutical, Nutritional and Sport Sciences (VDS PhaNuSpo), University of Vienna, Vienna, Austria; ^3^ Vienna Metabolomics Center (VIME), University of Vienna, Vienna, Austria; ^4^ College of Food Science and Technology, Huazhong Agricultural University, Wuhan, China; ^5^ Molecular Systems Biology (MOSYS), Department of Functional and Evolutionary Ecology, University of Vienna, Vienna, Austria

**Keywords:** sulforaphane, macrophages, M1 polarization, glycolysis, PKM2, interleukin 1 beta

## Abstract

Murine macrophages activated by the Toll-like receptor 4 agonist lipopolysaccharide (LPS) polarize to the M1 type by inducing proinflammatory marker proteins and changing their energy metabolism to increased aerobic glycolysis and reduced respiration. We here show that the aliphatic isothiocyanate sulforaphane (Sfn) diminishes M1 marker expression (IL-1β, IL-6, TNF-α, iNOS, NO, and ROS) and leads to highly energetic cells characterized by both high glycolytic and high respiratory activity as assessed by extracellular flux analysis. Focusing on a potential connection between high glycolytic activity and low IL-1β expression in M1 (LPS/Sfn) macrophages, we reveal that Sfn impedes the moonlighting function of pyruvate kinase M2 (PKM2) in M1 macrophages. Sfn limits mono/dimerization and nuclear residence of PKM2 accompanied by reduced HIF-1α levels, Stat3 phosphorylation at tyrosine 705, and IL-1β expression while preserving high levels of cytosolic PKM2 tetramer with high glycolytic enzyme activity. Sfn prevents glutathionylation of PKM2 in LPS-stimulated macrophages which may account for the reduced loss of PKM2 tetramer. Overall, we uncover PKM2 as a novel affected hub within the anti-inflammatory activity profile of Sfn.

## Introduction

Macrophages are cells of the innate immune system with a variety of functions, including combat of infections, initiation or resolution of inflammation, phagocytic clearance of debris, antigen processing and presentation to T-cells, as well as maintenance of tissue homeostasis, shortly they “SHIP,” i.e. sample, heal, inhibit, and present (1). In order to engage in such diverse and context-dependent activities, macrophages are highly plastic and adopt distinctive functional phenotypes or polarization states. There are the classically activated pro-inflammatory M1 type and the alternatively activated anti-inflammatory M2 type, representing only the extremes of a continuum of possible distinct intermediates. M1 macrophages are typically induced by lipopolysaccharide (LPS) alone or interferon (IFN)-γ/LPS, kill and clear pathogens, and show increased expression of proinflammatory markers, such as interleukin (IL)1-β, IL-6, tumor necrosis factor (TNF)-α, or inducible NO synthase (iNOS or NOS2), and produce microbicidal reactive oxygen species (ROS) and nitric oxide (NO). M2(a) macrophages are obtained after stimulation with IL-4 or IL-13 and participate in tissue remodeling, wound repair, and express transforming growth factor (TGF)-β, arginase 1, mannose receptor (Mrc1; CD206), IL-10, or C-type lectins (Mgl1/2) and produce polyamines (2, 3). Notably, macrophage polarization is accompanied and partly causally driven by characteristic shifts in their energy metabolism. Those allow a sufficient supply of needed ATP reducing equivalents and building blocks, as well as substrates for necessary posttranslational modifications or epigenetic tags. M1 macrophages stand out by the elevated activity of aerobic glycolysis (similar to the Warburg effect in cancer cells), a broken tricarboxylic acid (TCA) cycle with an accumulation of citrate, itaconate, and succinate, as well as the use of mitochondria for ROS rather than for ATP production. M2 macrophages usually show coupled oxidative phosphorylation (OXPHOS) with augmented fatty acid oxidation, an intact TCA cycle, or an increased hexosamine pathway (4–7).

Sulforaphane (Sfn), an aliphatic isothiocyanate found as a glucosinolate precursor in cruciferous vegetables, is highly investigated in the context of cancer prevention and inflammation-related disorders, including psoriasis, inflammatory bowel disease, neurodegeneration, or atherosclerosis (e.g., 8–13). Numerous *in vitro* and *in vivo* studies revealed the ubiquitin ligase adapter Keap1 as one of Sfn’s main targets (14, 15). Keap1, in turn, is an inhibitor (by triggering proteasomal degradation) of the cytoprotective transcription factor nuclear factor E2-related factor (Nrf)2, which is primarily involved not only in the adaptive homeostatic response to redox-, proteotoxic-, or xenobiotic insults but also in diminishing inflammation (16, 17). Intriguingly, activated Nrf2 has also an impact on cellular metabolism (18–21). The consolidated anti-inflammatory property of Sfn, the connection between activated Nrf2 and cellular bioenergetics, as well as the important role of metabolism for macrophage biology, prompted side-by-side examination of the influence of Sfn on murine macrophage polarization and bioenergetics as well as of their potential interdependence.

## Material and methods

### Reagents and chemicals

Stimuli for macrophage polarization, i.e., LPS from *Escherichia coli* O55:B5 (#L2880) and mouse IL-4 (#SRP3211), as well as Sfn (#S4441), TEPP-46 (#505487), FX11 (#427218), 2-deoxy-d-glucose (2-DOG) (#D8375), rotenone (rot) (#R8875), antimycin A (AA) (#A8674), oligomycin (#O4876), sodium pyruvate (#P5280), α-d-glucose (#158968), sulfanilamide (#S9251), and napthyl-ethylene-diamine (#8.06206) were purchased from Sigma Aldrich. Dimethyl sulfoxide (DMSO) (#M81802, Sigma, Sigma Aldrich, Vienna, Austria) was used as vehicle control and solvent for stock solutions of Sfn, TEPP-46, and other commonly used inhibitors. The DMSO concentration was even throughout the different conditions of one experiment and never exceeded a final concentration of 0.2%. 2′,7′-Dichlorodihydrofluorescein diacetate (H_2_DCFDA; #D399) was obtained from Invitrogen. Disuccinimidylsuberate (DSS) was provided by Thermo Fisher Scientific (#A39267). Media and supplements for cell culture were provided by Invitrogen or Lonza. Anti-PKM2 (#3198), anti-phospho-PKM2 (Tyr105) (#3827), anti-IL-1β (#12242), anti-HIF-1α (#14179), anti-phospho-Stat3 (Tyr705) (#9131), anti-α/β-tubulin (#2148), anti-lamin B1 (#12586S), anti-rabbit secondary horseradish-peroxidase (HRP)-labeled (#7074), and anti-mouse secondary HRP-labeled antibodies (#7076) were purchased from Cell Signaling Technologies, anti-actin antibody was from MP Bio (#0869100-CF), and the anti-glutathione (#sc-52399) antibody was obtained from Santa Cruz.

### Cultivation and treatment of cells

Immortalized bone marrow-derived macrophages (iBMDM) were kindly provided by Laszlo Nagy (Debrecen University, Hungary) and cultured in Dulbecco’s modified Eagle’s medium (DMEM)/high glucose supplemented with 10% filtered L-929 cell-conditioned medium containing macrophage colony-stimulating factor (M-CSF), 10% heat-inactivated FBS, 2 mM l-glutamine, 100 IU/ml penicillin, and 100 µg/ml streptomycin (iBMDM medium) at 37°C with 5% CO_2_. L-929-conditioned media were prepared by seeding L-929 cells (1.7 × 10^7^) in 40 ml of complete DMEM incubated at 37°C with 5% CO_2_ for 7 days. J774A.1 murine macrophages (LGC PromoChem) were cultured in DMEM/high glucose containing 10% heat-inactivated FBS, 2 mM l-glutamine, 100 IU/ml penicillin, and 100 µg/ml streptomycin (complete DMEM) at 37°C with 5% CO_2_. For polarization and treatment, iBMDM were seeded in iBMDM medium (10 × 10^4^ cells/well for 96-well plates, and 1.5 × 10^6^ cells/well for 6-well plates) 24 h prior to the experiments. The medium was then replaced with complete DMEM medium containing the desired test compounds and inhibitors, and cells were incubated for 30 min at 37°C. Polarization was initiated by the addition of LPS (25 ng/ml) and IL-4 (20 ng/ml) to obtain proinflammatory M1 (LPS) and anti-inflammatory M2 (IL-4) macrophages, respectively. J774A.1 cells were handled accordingly, except that they were routinely cultured in complete DMEM and exposed to 500 ng/ml LPS for successful M1 polarization. For a graphic depiction of the treatment protocol, please refer to **Supplementary Figure S1**. For the rest of the manuscript, we refer to LPS- and IL-4–treated macrophages as M1 (LPS) and M2 (IL4), respectively, and to macrophages pretreated with Sfn for 30 min and then exposed to LPS as M1 (LPS/Sfn).

### Assessment of nitric oxide/nitrite

Cells were seeded in 96-well plates and treated as indicated. An aliquot of the cell culture supernatant was then mixed 1:1 with the freshly prepared Griess reagent (0.5% sulfanilamide, 0.05% naphthyl-ethylene-diamine) and incubated for 10 min at room temperature. The absorbance was measured at a wavelength of 550 nm using a microplate spectrophotometer (TECAN Sunrise™ Austria).

### Assessment of intracellular reactive oxygen species

After the indicated treatments, cells were incubated with 20 µM of the ROS-sensitive dye 2′,7′-dichlorodihydrofluorescein diacetate (H_2_DCFDA) for 30 min at 37°C. After washing steps with PBS, fluorescence (geo mean after correction for autofluorescence) was monitored by flow cytometric analysis in the FL1H channel of a FACSCalibur™ (BD Biosciences).

### RNA extraction and qPCR analysis

Total RNA was extracted using an RNA isolation kit (#845-KS-2040250, IST Innuscreen GmbH) according to the manufacturer’s protocol. RNA integrity was routinely assessed by agarose gel electrophoresis, and quantification was achieved by spectrometric measurements using a NanoDrop 2000 (Thermo Fisher Scientific), also allowing a purity check *via* the A260/A280 ratio (≥2.0). cDNA was synthesized from 1 μg of RNA using a High-Capacity cDNA Reverse Transcription Kit (Thermo Fisher-#4368813), again following the manufacturer’s instructions. mRNA quantitation was performed using the Luna Universal qPCR Master Mix (#M3003E, New England Biolabs) and the LightCycler^®^480 Real-Time PCR System (Roche Diagnostics GmbH), using a cycling protocol of one denaturation step (10 min at 95°C) and up to 45 amplification cycles (15 s at 95°C, 30 s at 60°C) as well as melting curves between 55 and 95°C. The quality of the amplification was ensured by a single peak in the melting curve, only one amplicon of the desired size on an agarose gel, and no amplification in the negative (no template) control. Data analysis was performed by using the (2^–ΔΔCt^) method. Used gene-specific primers were designed with the help of Primer3 and BLAST (for sequences, see **Table 1**) and ordered from Thermo Fisher Scientific. Their amplification efficiencies were confirmed to range between 95.0% and 100% under our experimental conditions. *ppia* was taken as a reference gene as it excelled in pilot experiments over other tested reference genes (*actin*, *18S*, *stx5*, or *hprt1*) in the used experimental setups due to its stable expression throughout the employed treatment regimens and its Cq values close to the ones of the investigated target genes.

**Table 1:** List of qPCR primers (5’-3’).

*mrc1*	*Fwd: CTCTGTTCAGCTATTGGACGC* *Rev: CGGAATTTCTGGGATTCAGCTTC*
*arg1*	*Fwd: TTTTAGGGTTACGGCCGGTG* *Rev: CCTCGAGGCTGTCCTTTTGA*
*mgl1*	*Fwd: TGCAACAGCTGAGGAAGGACTTGA* *Rev: AACCAATAGCAGCTGCCTTCATGC*
*mgl2*	*Fwd: GCATGAAGGCAGCTGCTATTGGTT* *Rev: TAGGCCCATCCAGCTAAGCACATT*
*cd36*	*Fwd: GAGCAACTGGTGGATGGTTT* *Rev: GCAGAATCAAGGGAGAGCAC*
*nos2*	*Fwd: CAGAGGACCCAGAGACAAGC* *Rev: TGCTGAAACATTTCCTGTGC*
*il6*	*Fwd: GAGGATACCACTCCCAACAGACC* *Rev: AAGTGCATCATCGTTGTTCATACA*
*il1β*	*Fwd: CAACCAACAAGTGATATTCTCCATG* *Rev: GATCCACACTCTCCAGCTGCA*
*ppia*	*Fwd: CCAAGACTGAATGGCTGGATG* *Rev: TGTCCACAGTCGGAAATGGTG*

### Assessment of polyamine synthesis, TGF-β− or TNF-α secretion

After treatment as indicated, the total polyamine content was measured by the Total Polyamine Assay Kit from Biovision (#K475-100). Cytokine release (TNF-α and TGF-β) was assessed using the Mouse Tumor Necrosis Factor α ELISA Kit (# RAB0477, Sigma Aldrich) or Transforming Growth Factor β-1 Mouse ELISA Kit (#BMS608-4, Invitrogen).

### Protein extraction, crosslinking, and Western blot analysis

Total cell lysates were obtained by incubation of washed cells with NP40 lysis buffer (150 mM NaCl, 1% NP40, 10 mM DTT in 50 mM Tris (pH 8.0)) with a mixture of protease and phosphatase inhibitors on ice for 10 min. Cell lysates were then scraped from the culture plates, followed by short sonication. After centrifugation for 15 min (11,000×*g* at 4°C), supernatants containing total protein were collected. For fractionation of nuclear and cytosolic proteins, the NE-PER nuclear and cytoplasmic extraction kit (#78833, Thermo Fisher Scientific) was used following the manufacturer’s instructions. For assessing mono/di/tetramers of PKM2, 5 mM of the crosslinker DSS was added to the cells for 30 min prior to quenching with Tris-HCl (pH 7.5) to a final concentration of 20 mM for 15 min at room temperature and subsequent lysis. The protein concentration of the samples was determined by the ROTI^®^Quant (#K0151, Carl Roth) protein assay. Equal amounts of protein were separated on 7.5% or 10% sodium dodecyl sulfate-polyacrylamide gels by denaturing electrophoresis (SDS-PAGE) and transferred to a 0.2-μm polyvinylidene difluoride membrane (PVDF). The membranes were blocked with 5% bovine serum albumin (BSA) in TBST for 1 h at room temperature and incubated with specific primary antibodies at 4°C overnight (antibody dilution 1:1,000 in TBST), followed by HRP-conjugated secondary antibody incubation at room temperature for 1 h. Signal intensity was measured using enhanced chemiluminescence (ECL) and an Amersham ImageQuant 800 imager (Cytiva). The densitometric analysis of the bands was measured using proprietary Cytiva or ImageJ software.

### Immunoprecipitation

For assessment for glutathionylated PKM2, cell lysates were prepared under nonreducing conditions and incubated with anti-GSH antibody (dilution 1:100) at 4°C overnight. The lysates were then added to washed magnetic A/G sepharose beads (#88802, Pierce) and rolled end-over-end for 2 h at room temperature. Beads were then thoroughly washed, and bound antigen/antibody complexes were eluted by incubation with SDS sample buffer (again, no reducing agent) at room temperature for 10 min. Subsequent immunoblot analysis followed the protocol described above.

### Extracellular flux analysis

Cells were seeded and treated as desired. On the day of measurement, cells were scraped and plated (1 × 10^5^ cells/well) in XF24e-cell culture plates precoated with Corning^®^ Cell-Tak adhesive (#CLS354240, Sigma Aldrich). Cells were incubated in an XF assay medium (pH 7.4/37°C) (#103575-100, Agilent Technologies) supplemented with 2 mM glutamine, 1 mM pyruvate, and 25 mM glucose for 1 h at 37°C in a non-CO_2_ incubator. Real-time extracellular acidification (ECAR) and oxygen consumption (OCR) rates were measured in an XF24e Flux Analyzer (Seahorse Bioscience-Agilent Technologies). ECAR and OCR were monitored under basal conditions (as well as after rotenone (Rot, 0.5 µM)/antimycin A (AA; 0.5 µM) and 2-DOG injections (50 mM), using the proprietary glycolytic rate assay protocol from Agilent (#103344-100). Data were analyzed using the Wave software package from Agilent Technologies.

### GC/MS-based analysis of citric acid, itaconic acid, and succinic acid

iBMDM (5 × 10^5^ cells/well) were washed with 2 ml of warm 0.9% NaCl and extracted in 1 ml precooled (−80°C) 80% MeOH. A total of 5 µl of an internal standard mixture (norvaline, 10 mM; norleucine, 1.25 mM; phenyl β-d-glucopyranoside, 1.25 mM; raffinose, 1.25 mM) was added. Samples were shaken at 700 rpm for 15 min at 4°C and then centrifuged at 21, 000×*g* for 10 min at 4°C. In total, 450 µl of the supernatant was transferred and dried in a vacuum concentrator (ScanVac, LaboGene) with a stepwise gradient. Samples were stored at −80°C until further processing. Derivatization was started by the addition of 20 µl of methoxyamine hydrochloride solution (40 mg*ml^−1^ pyridines) to the pellet and incubation for 90 min at 30°C and 700 rpm. For silylation, 80 µl of *n*-methyl-*N*-trimethylsilyltrifluoracetamide (MSTFA) was added and shaken for 30 min at 37°C and 750 rpm. The samples were centrifuged at 14,000×*g* for 4 min and 24°C. Furthermore, 70 µl of the supernatant was transferred to glass vials with inserts and sealed with crimp caps. Even-numbered *n*-alkanes (C10–C40) were measured with each batch for retention index calculation. Metabolites were analyzed utilizing gas chromatography and time-of-flight mass spectrometry on a Pegasus BT GC-TOF-MS (LECO Corporation, St. Joseph, MI, USA), according to a previously established method with modifications (22). Each batch consisted of a quality control mix, samples, blanks, and an alkane standard. Samples were separated by gas chromatography (7890B, Agilent, Santa Clara, CA, USA) on an Agilent column HP5MS (30 m length, 0.25 mm diameter, 0.25 µm film; Agilent Technologies, Santa Clara, USA) with a flow rate of 1 ml helium/min in split-less mode. The following settings were used: injection temperature of 230°C, transfer line of 250°C, temperature gradient as follows: 70°C for 1 min, then increase by 9°C/min up to 340°C and hold for 15 min. Metabolites were recorded on a BT-TOF mass spectrometer (LECO Corporation) with an acquisition rate of 10 spectra/s, a mass range of 50 to 550, and an ion source temperature of 250°C. Files were deconvoluted and processed using the software ChromaTOF (LECO Corporation, version 5.51.50.0.68774) and matched to an in-house reference library. The metabolite data set was normalized to the internal standard for the corresponding split measurement with the minimum retention index difference and the cell number for each condition with two replicates, performed in a parallel experiment. ANOVA, Tukey’s table, and boxplots were performed using the online tool Metaboanalyst v5.0 with the following settings: no data filtering, log transformation (base 10), and autoscaling.

### Statistics

Unless stated otherwise, at least three independent biological replicates were performed for the experiments. The bar graphs depict the mean ± standard deviation (SD). Groups were compared *via* ANOVA and multiple-comparisons tests by using GraphPad Prism 6 software. Differences were considered as significant if *p* < 0.05.

## Results

### Sfn impedes M1 marker expression in LPS-treated iBMDM

**Figure 1:**
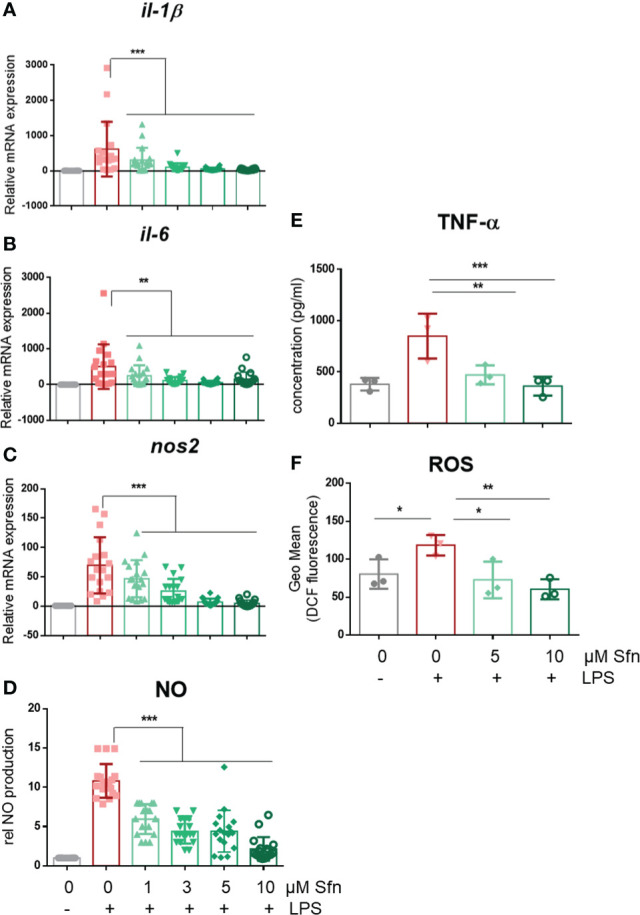
Sfn impedes M1 marker expression during macrophage polarization initiated by LPS. IBMDM macrophages were pretreated with DMSO or the indicated concentrations of Sfn for 30 min before they were stimulated with LPS (25 ng/ml) for 24 h. RNA was extracted, reversely transcribed, and subjected to qPCR analysis for *il1β* **(A)**, *il6* **(B)**, and *nos2* **(C)** mRNA expression. *Ppia* served as a reference gene; data were referred to as M0 controls. Secretion of TNF-α **(D)** was assessed using an ELISA, and nitric oxide **(E)** was measured by monitoring nitrite levels *via* the Griess assay as described in the *Methods* section. Levels of intracellular ROS **(F)** were determined by H_2_DCF-DA staining and subsequent flow cytometric analysis. Data depict mean ± SD from at least three independent biological replicates (^*^ *p* < 0.05, ^**^ *p* < 0.01, ^***^ *p* < 0.001; ANOVA, followed by multiple comparisons test).

**Figure 2:**
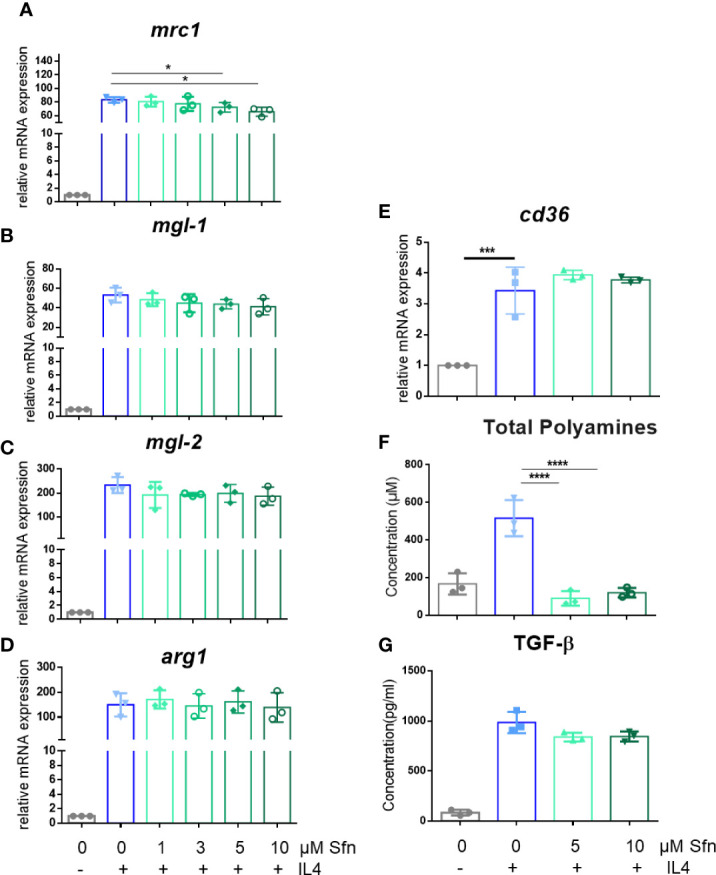
Sfn does not markedly interfere with M2 marker expression during macrophage polarization initiated by IL-4. IBMDM macrophages were pretreated with DMSO or with the indicated concentrations of Sfn for 30 min before they were stimulated with IL-4 (20 ng/ml) for 24 h. RNA was extracted, reversely transcribed, and subjected to qPCR analysis for *mrc1* **(A)**, *mgl1* **(B)**, *mgl2* **(C)**, *arg1* **(D)**, and *cd36* **(E)** mRNA expression. *Ppia* served as a reference gene; data were referred to as M0 controls. Production of total polyamines **(F)** was assessed by a commercial kit and TGF-β **(G)** by an ELISA as described in the *Methods* section. Data depict mean ± SD from at least three independent biological replicates (^*^ *p* < 0.05; ***p < 0.001;^****^ *p* < 0.0001; ANOVA, followed by multiple comparisons test).

In a first step, we investigated the influence of Sfn during M1 (LPS) and M2 (IL4) polarization of iBMDM. For all experiments, Sfn concentrations were between 1 and 10 µM, which showed no negative impact on the viability of differentiated iBMDM (**Supplementary Figure S2**) after 24 h of incubation. In line with previous reports (23–25), the presence of Sfn led to a significant reduction of M1 traits in LPS-stimulated iBMDM: it significantly reduced mRNA expression of *ilβ*, *il6*, and *nos2*, diminished TNF-α release, and reduced ROS and NO production (**Figure 1**). Concerning M2 (IL4) polarization, Sfn did not elicit any (*mgl1*, *mgl2*, *arg1*, *cd36*) or very minor (*mrc1*) inhibition or activation of M2 marker mRNA expression or TGF-β secretion (**Figure 2**). It significantly blunted only polyamine synthesis, a typical M2 response downstream of arginine conversion to urea (**Figure 2**). This may be due to the reported inhibition of ornithine decarboxylase, an enzyme in the polyamine synthesis pathway (26), which, however, awaits experimental proof in our system. Sfn had no impact on investigated markers in naïve macrophages (**Supplementary Figure S3**).

### M1(LPS/Sfn) iBMDM show an intact TCA cycle and both high glycolytic and respiratory activities

**Figure 3:**
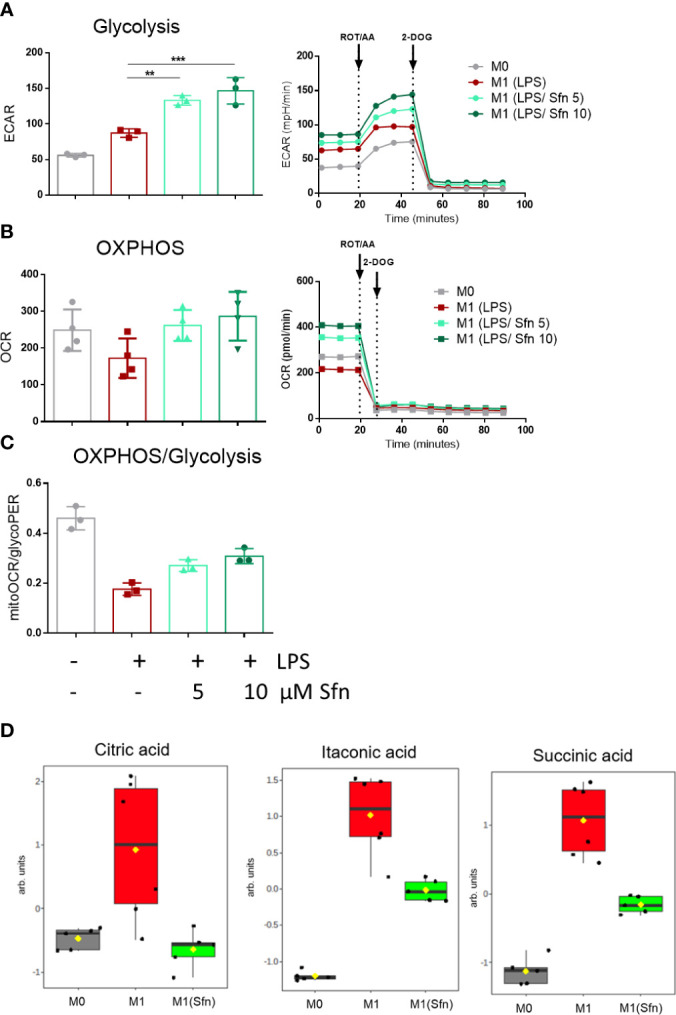
Sfn leads to high glycolytic and respiratory activities and blunts TCA cycle breaks in M1 (LPS) macrophages. IBMDM macrophages were pretreated with DMSO or with the indicated concentrations of Sfn for 30 min before they were stimulated with LPS (25 ng/ml) for 24 h. A total of 100,000 cells were then subjected to glycolytic rate assay and extracellular flux analysis as described in detail in the *Methods* section. **(A)** Depicts glycolytic activity (as evident in extracellular acidification rate (ECAR) in mPH/min), **(B)** OXPHOS activity (as evident oxygen consumption rate (OCR) in pmol/min), and **(C)** the ratio between basal mitoOCR/glycoPER. Bar graphs depict compiled data of basal ECAR and/or OCR from three different biological replicates given as mean ± SD. (^**^ *p* < 0.01; ^***^ *p* < 0.001; ANOVA, followed by multiple comparisons test). Graphs on the right show exemplary data from the performed glycolytic rate assays. **(D)** MS-based determination of citrate, itaconate, and succinate levels in M0, M1 (LPS), and M1 (LPS/Sfn) after 24 h incubation ([Sfn] = 10 µM). Graphs depict compiled data from at least five independent biological replicates (arb. units, arbitrary units; detailed information can be found in the *Methods* section).

In the next step, selected metabolic features were assessed by extracellular flux and MS-based metabolite analyses. First, we successfully confirmed expected bioenergetic changes during polarization to M1 (glycolysis^high^/OXHOS^low^) and M2 (glycolysis^low^/OXHOS^high^) macrophages under the used iBMDM cultivation and treatment conditions (**Supplementary Figure 4**). As the preceding functional marker analysis had uncovered that Sfn mainly affected M1 polarization, we continued to compare metabolic features of M0, M1 (LPS), and M1 (LPS/Sfn). Judged against M0 and M1 (LPS), M1 (LPS/Sfn) showed elevated glycolytic activity as evident in higher ECAR values (**Figure 3A**). Sfn also enabled macrophages to maintain higher OXPHOS rates after LPS stimulation than seen in control M1 (**Figures 3B, C**). Sfn was able to blunt the breaks in the TCA cycle, which were obvious by increased levels of citrate, itaconate, and succinate after 24 h in M1 (LPS) (**Figure 3D**).

To sum up, Sfn showed no marked impact on M2 polarization but impeded expression of proinflammatory M1 markers, favored an intact TCA cycle, and preserved OXPHOS with concomitant high glycolytic activity in LPS-treated macrophages.

### Interference with glycolysis does not attenuate the inhibitory effect of Sfn on IL-1β expression

**Figure 4:**
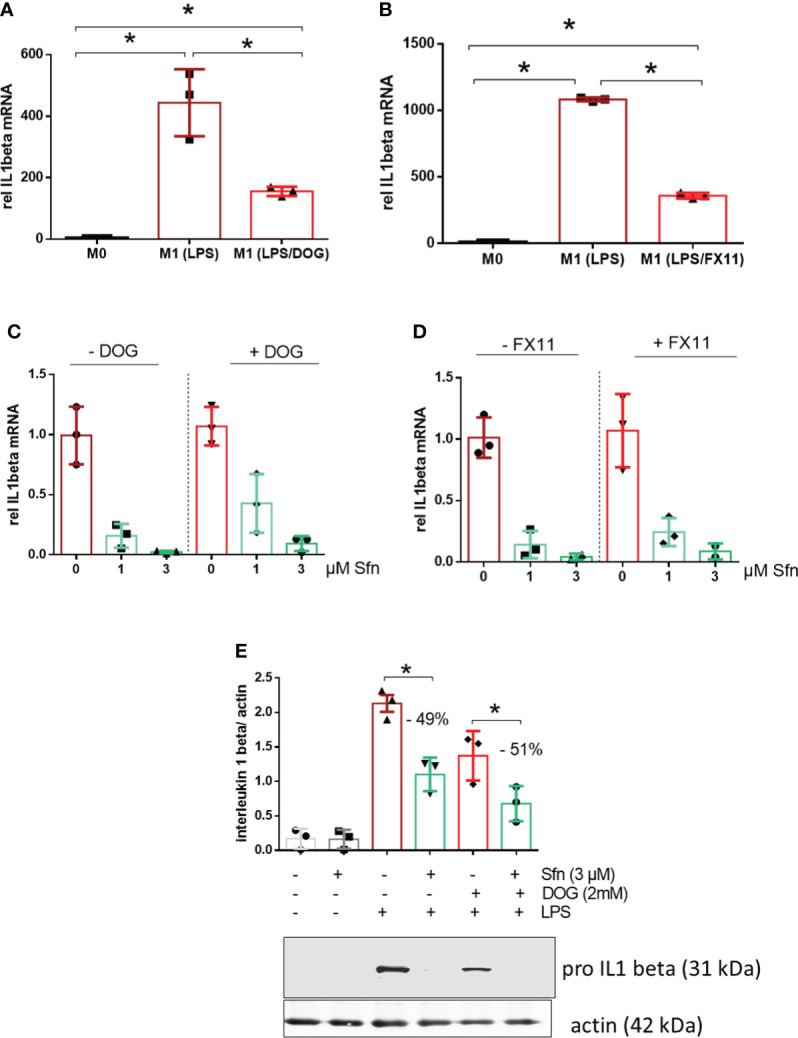
DOG or the LDH inhibitor FX11 cannot attenuate inhibition of IL-1β expression by Sfn. iBMDM were left untreated (M0), treated with LPS (25 ng/ml) (M1) and **(A)** LPS + DOG (2 mM) or **(B)** LPS + FX11 (10 µM) for 6 h before relative mRNA expression of IL-1β was assessed by qPCR (using *ppia* as reference gene). iBMDM were pretreated with DMSO or Sfn (1 or 3 µM) for 30 min in the absence or presence of either **(C)** 2 mM (DOG) or **(D)** 10 µM FX11 before LPS (25 ng/ml) was added for 6 h. Then mRNA expression of *il1β* was assessed by qPCR (using *ppia* as reference gene) and referred to the respective DMSO controls. **(E)** Murine macrophages were pretreated with DMSO or Sfn (3 µM) for 30 min in the absence or presence of 2 mM DOG before LPS was added for another 6 h. After protein extraction total cell lysates were then subjected to immunoblot analysis for pro-IL-1β and actin as a loading control. Representative blots are shown and bar graphs depict compiled densitometric data from three biological replicates (mean ± SD, *n* = 3; ^*^ *p* < 0.05, ANOVA).

Notably, the expression of IL-1β is highly dependent on and driven by increased aerobic glycolysis during M1 polarization (27–29). Therefore, the observation of increased glycolytic rate and parallelly inhibited IL-1β expression in Sfn-treated M1 (LPS) macrophages was somewhat counterintuitive. In order to assess whether elevated glycolytic activity truly contributed to the inhibition of IL-1β expression by Sfn, DOG as an inhibitor of hexokinase or FX11 as an inhibitor of lactate dehydrogenase (at submaximal inhibitory concentrations to impede but not completely wipe out glycolysis and M1 polarization) were co-administered to M1 macrophages. As expected, the glycolytic inhibitors reduced *il1β* mRNA expression upon LPS stimulation (**Figures 4A, B**), but they could not markedly weaken the anti-inflammatory potential of Sfn (**Figures 4C, D**), as evident in comparable inhibition rates in the presence and absence of glycolytic inhibitors. A similar picture was observed when examining protein levels of pro-IL-1β: DOG alone already reduced expression of the cytokine precursor but could not diminish the relative inhibition by Sfn (**Figure 4E**). These data suggest that the observed increased glycolytic activity per se was not essential but rather a bystander in the inhibition of IL-1β expression upon Sfn exposure.

### M1 (LPS/Sfn) displays reduced HIF-1α abundance and Stat3 (Y705) phosphorylation accompanied by reduced dimerization, nuclear translocation, and glutathionylation of PKM2

**Figure 5:**
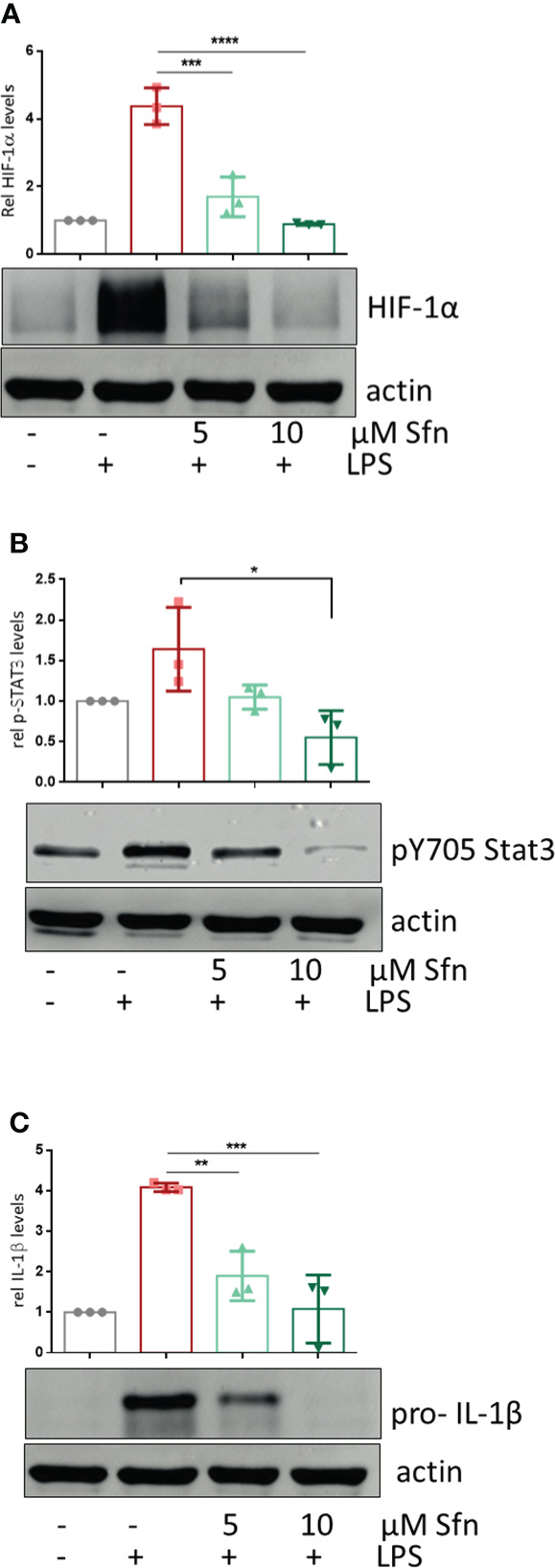
Sfn leads to diminished HIF-1α abundance, Stat3 (Y705) phosphorylation, and pro-IL-1β expression. iBMDM were pretreated with DMSO or Sfn at the indicated concentrations for 30 min prior to stimulation with LPS (25 ng/ml). After 6 h, cells were lysed and total cell lysates were subjected to immunoblot analysis for HIF-1α **(A)**, p(Y705) Stat3 **(B)**, pro-IL1beta **(C)**, and actin as a loading control. Representative blots are shown, and bar graphs depict compiled densitometric data from three biological replicates (mean ± SD, *n* = 3; ^*^ *p* < 0.05, ^**^ *p* < 0.01, ^***^ *p* < 0.001, and ^****^ *p* < 0.0001; ANOVA, followed by multiple comparisons test).

**Figure 6:**
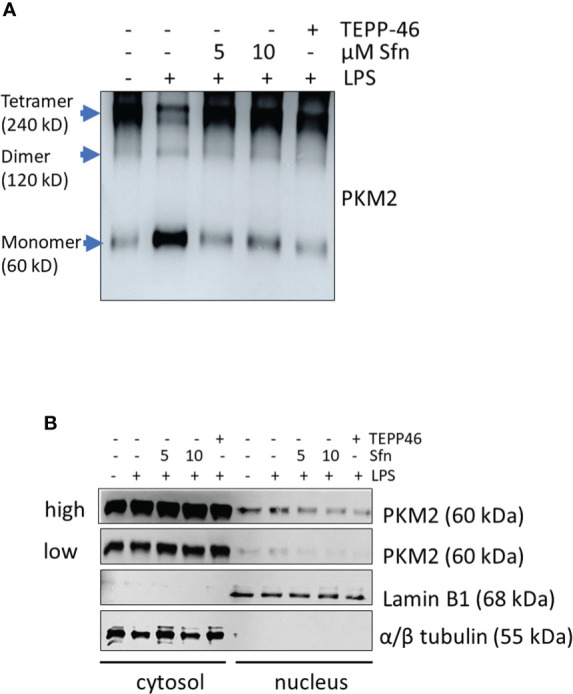
Sfn prevents tetramer loss and nuclear localization of PKM2 in M1 (LPS) macrophages. iBMDM were pretreated with DMSO, Sfn at the indicated concentrations or TEPP-46 (20 µM) for 30 min prior to stimulation with LPS (25 ng/ml) for 6 h. **(A)** Cellular proteins were then crosslinked with DSS as described in the *Methods* section and harvested. Lysates were subjected to immunoblot analysis for PKM2. **(B)** Cytosolic and nuclear protein fractions were prepared and subjected to immunoblot analysis for PKM2 and lamin or tubulin as a nuclear or cytosolic loading control. Representative blots from three biological replicates with consistent results are depicted. (*high* and *low* exposure times for the PKM2 blots are shown to account for different signal strengths between cytosolic and nuclear fractions).

**Figure 7:**
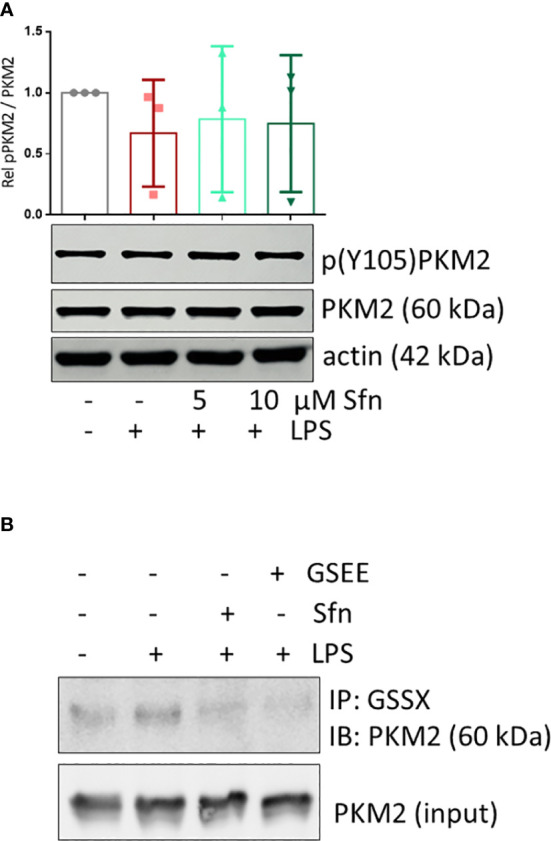
Sfn does not affect Y105 phosphorylation but glutathionylation of PKM2 in M1 (LPS) macrophages. iBMDM were pretreated with DMSO or the indicated concentrations of Sfn for 30 min prior to stimulation with LPS for 6 h. **(A)** Total cell lysates were immunoblotted for p(Y105), total PKM2, and actin as a loading control. Representative blots of at least three independent experiments are depicted. Bar graphs represent compiled densitometric data. **(B) **Macrophages were pretreated with Sfn (10 μM) or the GSH donor glutathioneethylester (GSEE) before LPS was added for another 6 h. Proteins were harvested under nonreducing conditions. Lysates (input) or immunoprecipitates obtained with an antibody specific for protein-bound glutathione (GSS-X) were subjected to immunoblot analysis for PKM2. Representative blots of three independent experiments with consistent results are depicted.

To shed more light on mechanisms beyond increased glycolytic activity in M1 (LPS/Sfn), cellular glucose uptake rates and expression of glycolytic enzymes were further examined. Whereas LPS stimulation induced a significant increase in the glucose uptake rate, there was no significant difference between M1 (LPS) and M1 (LPS/Sfn) macrophages (**Supplementary Figure S5A**). Moreover, Sfn did not markedly alter the expression of key glycolytic enzymes in M1 macrophages, including hexokinase (HK) 1, HK2, phosphofructokinase (PFK)1, PFK2, or pyruvate kinase (PK) M2 (**Supplementary Figure S5B**). Regarding the latter, it is noteworthy that PKM2 has—next to its canonical enzymatic activity in glycolysis—moonlighting, i.e., nonmetabolic functions. As a tetramer, the enzyme resides in the cytosol and shows high glycolytic enzymatic activity in converting phosphoenolpyruvate to pyruvate. As a monomer/dimer, it shows reduced enzymatic activity in glycolysis but can phosphorylate and activate proteins such as signal transducer and activator of transcription (Stat)3, and translocate to the nucleus, where it can act as a stabilizing scaffold for hypoxia-inducible factor (HIF)-1α, thus driving expression of genes, including *il1β* (30–32). Focusing on the nuclear PKM2-HIF-1α/Stat3-IL-1 axis in M1 (LPS/Sfn), immunoblots revealed that Sfn was able to block Stat3 phosphorylation, HIF-1α stabilization, and pro-IL-1β expression in M1 macrophages (**Figures 5A–C**). Moreover, compared to control M1 (LPS) cells, tetramerization of PKM2 was obviously preserved in M1 (LPS/Sfn) or M1 (LPS/TEPP-46) macrophages (the known glycolytic PKM2 activator TEPP-46 was used as positive control) (**Figure 6A**). Consistently, nuclear PKM2 showed elevated signals in M1 (LPS) macrophages which were markedly lowered in the presence of Sfn or TEPP-46 (**Figure 6B**). Impeded tetramerization can be driven by binding to tyrosine-phosphorylated peptides or various posttranslational modifications of PKM2, including phosphorylation at tyrosine 105 or modification of cysteine residues by disulfide formation with glutathione (33–35). Examination of phospho- and total PKM2 did not uncover any consistent differences between the investigated samples after an incubation of 6 h (**Figure 7A**). However, glutathionylated PKM2 (detected *via* immunoprecipitation of protein-bound glutathione and subsequent immunoblot for PKM2) reproducibly gave a stronger signal for control M1 (LPS) than for M1 macrophages treated with Sfn (**Figure 7B**). These data may indicate that Sfn counteracts glutathionylation and dimer formation of PKM2. Like this, Sfn on the one hand may maintain tetramer formation and high enzymatic activity of PKM2 in glycolysis, and on the other hand, it reduces nuclear PKM2 abundance and IL-1β expression *via* Stat3 and/or HIF1. Notably, examining cellular ratios of reduced glutathione and glutathione disulfide (GSH/GSSG) (**Supplementary Figure S6**) 6 h after treatment showed lower values in M1 (LPS/Sfn) compared to M1 (LPS), possibly suggesting limited substrate (GSH) supply for PKM2 modification in the presence of Sfn.

## Discussion

Compared to control M1 (LPS), M1 (LPS/Sfn) macrophages showed (i) reduced expression of typical M1 markers, including IL-1β, and (ii) both high glycolytic and high respiratory activity as well as an unbroken TCA cycle. The increased cellular glycolytic activity *per se* (iii) apparently was not essential for the inhibitory action of Sfn on IL-1β expression but (iv) coincided with reduced PKM2 glutathionylation and elevated levels of tetrameric highly active PKM2. (v) The concomitantly reduced levels of (nuclear) PKM2 mono/dimer with nonmetabolic function were the ones to be followed by less HIF abundance, Stat3 tyrosine phosphorylation, and blunted IL-1β expression.

This study was driven by the notions that macrophage polarization might be fine-tuned by interfering with cellular metabolism (36) and that bioactive natural products may metabolic targets affect for eliciting bioactivity (37, 38). We, therefore, set out to dissect a potential causative relation between increased glycolytic activity and decreased IL-1β expression in Sfn-treated M1 macrophages. Although not within the scope of this study, the crucial role of mitochondria or OXPHOS in macrophage biology (39–40) deserves further attention in the context of a potential immunometabolic action of Sfn. This is particularly noteworthy given that activated Nrf2, a well-accepted target of Sfn, is known to affect mitochondrial function (41–43) and M1 (LPS/Sfn) macrophages already seemed to favor an intact TCA cycle and higher respiratory activity compared with control M1 (LPS) (see **Figure 3**).

Based on available knowledge, the assumption of a direct causal connection between the diminished IL-1β and elevated glycolytic activity appeared daring, as a switch to increased glycolysis had commonly been described as proinflammatory and essential to drive IL-1β expression (29, 44, 45). Experiments with two different pharmacological glycolytic inhibitors indeed strongly suggested that the unhindered flow of intermediates through the glycolytic reactions was dispensable and not the prime explanation for the inhibitory effect of Sfn on IL-1β mRNA and protein levels. Rather, the observed increased extracellular acidification seemed to occur collaterally to an impeded moonlighting function of PKM2. That is to say, Sfn decreased nuclear mono/dimeric PKM2 and increased levels of highly metabolically active PKM2 tetramer in the cytosol compared to control M1 (LPS). Hence, cytosolic conversion of phosphoenolpyruvate to pyruvate (and then lactate) was increased (and measurable as increased ECAR) and the nuclear PKM2/Stat3-HIF axis/IL-1β was reduced, as seen in **Figures 3**, **5**. Notably, Sfn did not significantly alter M2 traits of murine macrophages, as observed for other PKM2 activators, such as TEPP-46 or DASA-58 (31), suggesting additional targets for Sfn. One conceivable candidate in this context could be polyamine synthesis which was inhibited by Sfn (see **Figure 2**) and uncovered as an important step in alternative M2 macrophage activation as well as T-cell differentiation (46–48). However, Sfn has also been reported to promote M2 polarization in human PBMC-derived M0 and M1 macrophages *in vitro* (49) or in microglial cells when fed to living rats (50). Hence, further investigations are necessary to completely dissect the influence of Sfn on M2 macrophage polarization or plasticity with an attentive eye on species- and context-dependent differences. A recent study also revealed that elevated glycolysis early on during macrophage M1 polarization dampened *il6* expression by restricting acetyl-coenzyme A for histone acetylation and subsequent gene expression (29). Given that Sfn also reduced the expression of *il6* and other proinflammatory markers in this study (see **Figure 1**), it would be interesting to examine whether any of these observations may be truly reliant on the increase in glycolytic metabolism in M1 (LPS/Sfn). It becomes more and more obvious that different M1 markers show distinct susceptibility to metabolic changes during polarization, also dependent on the timing of their expression during the inflammatory process (early vs. late phase genes). This renders immunomodulation by metabolic changes a highly gene- and time-sensitive endeavor in which causality needs to be clearly differentiated from mere correlation.

Impeding nuclear PKM2 may not be the only mechanism by which Sfn interferes with IL-1β expression. Being an electrophilic natural product with pronounced polypharmacology, Sfn has been reported to, e.g., inhibit nuclear factor (NF)-κB (23), activate transcription factor (TF) EB (51), or strongly induce Nrf2 signaling (52), which all could directly or indirectly contribute and sum up to the observed downregulation of proinflammatory markers. Additionally, we also uncovered reduced succinate levels in M1 (LPS/Sfn) (see **Figure 3**), which could contribute to reduced HIF stabilization as well (53). To what extent the diminished nuclear PKM2 dimer finally contributes to the reduced expression of IL-1β by Sfn or whether reduced glutathionylation is the only explanation for maintained tetramer formation still needs to be clarified. A boost in protein glutathionylation with the use of diamide (54) restored PKM2 glutathionylation in M1 (LPS/Sfn) and significantly interfered with Sfn’s capacity to reduce IL-1β expression (**Supplementary Figure S7A, B**), which would be in line with PKM2 modification as one involved step in the immunomodulation by Sfn. However, future more target-specific approaches, e.g., based on the determination of the exact sites of glutathionylation or other PTMs in PKM2, subsequent site-directed mutagenesis, and investigation of the influence of Sfn on macrophages carrying the mutant forms of PKM2, need to follow in order to unambiguously solve this issue. Involvement of Nrf2 in the PKM2 axis is plausible, as Sfn knowingly leads to Nrf2 stabilization (see also **Supplementary Figure S8**) and reduced PKM2 glutathionylation (see **Figure 7**) in our cell system, and Nrf2 is a prime regulator of glutathione pathways (and glycolysis) in macrophages (41). To explicitly corroborate this hypothesis, macrophages with an Nrf2 knockdown should be investigated with regard to PKM2 dimerization and IL-1β expression upon LPS + Sfn treatment. Interestingly, recent studies suggest PKM2-mediated transactivation of Nrf2 in neurons (55, 56), starting to paint a picture of complex mutual crosstalk between Nrf2 and PKM2.

Our study used bulk cultures of murine macrophages in *in vitro* culture and assessed a limited set of readouts at a few time points. Such snapshots, by definition, cannot fully capture the dynamic behavior of metabolism or the functional properties of single and plastic macrophages, miss feed-back and feed-forward loops, metabolite fluxes over time, impact of different oxygen tensions on metabolism or signaling, as well as the fuel competition and crosstalk that takes place between neighboring cells in living organisms. Future studies with multiomics approaches and isotope-labeled flux analyses on a single cell level or even metabolic analyses *in vivo* may give deeper and more comprehensive insights (57). Despite the mentioned limitations and gaps, this work could, for the first time, uncover moonlighting of PKM2 to be affected by Sfn in LPS-stimulated macrophages and may also be relevant for other settings. Likewise, modulation of PKM2 has already been exploited by various small molecules to mediate potential health benefits (e.g., 58–62). Staying in the field of immunology, Sfn-mediated interference with mono/dimer formation of PKM2 should be examined during Th1 or Th17 differentiation of T-helper cells (63–65), especially as Sfn already showed promise for diseases with a strong Th17 component, such as psoriasis (66) or arthritis (67). The full impact of Sfn on immunometabolism is only beginning to emerge and very likely warrants some exciting additions to the activity profile of this natural product.

## Data availability statement

The raw data supporting the conclusions of this article will be made available by the authors, without undue reservation.

## Author contributions

SB and EH conceived the study and designed experiments. SB, MB, FY, and EH performed experiments and analyzed and interpreted obtained data. EH provided funding. EH and WW supervised the study. SB and EH drafted the manuscript. All authors contributed to the article and approved the submitted version.

## Funding

This work was financially supported by the FWF (Austrian Science Fund; P32600 to EH) and the University of Vienna. FY is supported by a CSC fellowship.

## Acknowledgments

The authors thank Prof. Lazlo Nagy for generously providing iBMDM, Ana Rita Guimarães Machado Vaz da Silva and Marian Peteri for hands-on help with experiments during their internships, Barbara Braunböck-Müller, Scarlet Hummelbrunner, and Daniel Schachner for excellent technical assistance as well as the members of Molecular Targets Group for fruitful discussions.

## Conflict of interest

The authors declare that the research was conducted in the absence of any commercial or financial relationships that could be construed as a potential conflict of interest.

## Publisher’s note

All claims expressed in this article are solely those of the authors and do not necessarily represent those of their affiliated organizations, or those of the publisher, the editors and the reviewers. Any product that may be evaluated in this article, or claim that may be made by its manufacturer, is not guaranteed or endorsed by the publisher.
