# Corrigendum: Sulforaphane diminishes moonlighting of pyruvate kinase M2 and interleukin 1β expression in M1 (LPS) macrophages

**DOI:** 10.3389/fimmu.2024.1395642

**Published:** 2024-04-22

**Authors:** Sheyda Bahiraii, Martin Brenner, Fangfang Yan, Wolfram Weckwerth, Elke H. Heiss

**Affiliations:** ^1^ Department of Pharmaceutical Sciences, University of Vienna, Vienna, Austria; ^2^ Vienna Doctoral School of Pharmaceutical, Nutritional and Sport Sciences (VDS PhaNuSpo), University of Vienna, Vienna, Austria; ^3^ Vienna Metabolomics Center (VIME), University of Vienna, Vienna, Austria; ^4^ College of Food Science and Technology, Huazhong Agricultural University, Wuhan, China; ^5^ Molecular Systems Biology (MOSYS), Department of Functional and Evolutionary Ecology, University of Vienna, Vienna, Austria

**Keywords:** sulforaphane, macrophages, M1 polarization, glycolysis, PKM2, interleukin 1 beta

In the published article, there was an error in the legend for **Figure 7**, panel B as published. The labeling of the lanes in the immunoblots was incorrect.

**Figure 7:**
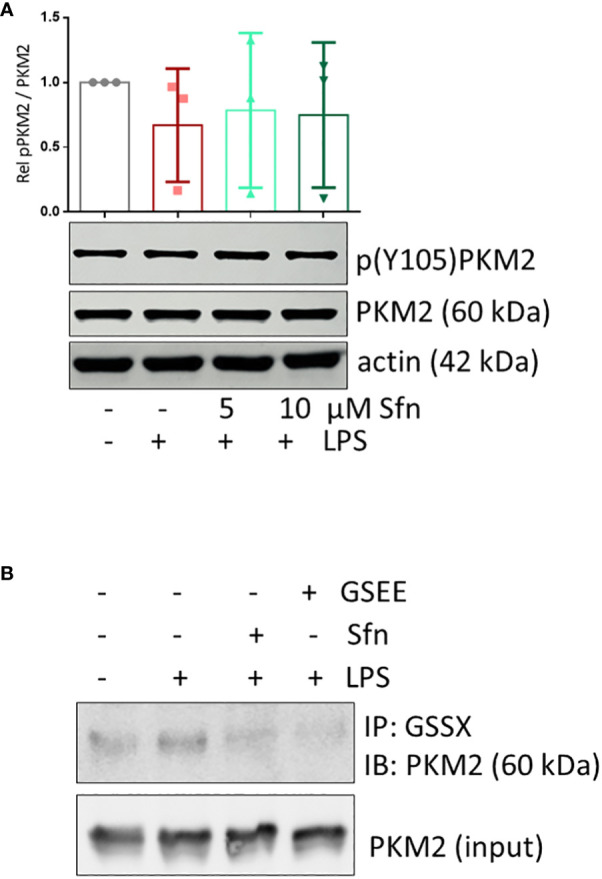
Sfn does not affect Y105 phosphorylation but glutathionylation of PKM2 in M1 (LPS) macrophages. iBMDM were pretreated with DMSO or the indicated concentrations of Sfn for 30 min prior to stimulation with LPS for 6 h. **(A)** Total cell lysates were immunoblotted for p(Y105), total PKM2, and actin as a loading control. Representative blots of at least three independent experiments are depicted. Bar graphs represent compiled densitometric data. **(B)** Macrophages were pretreated with Sfn (10 μM) or the GSH donor glutathioneethylester (GSEE) before LPS was added for another 6 h. Proteins were harvested under nonreducing conditions. Lysates (input) or immunoprecipitates obtained with an antibody specific for protein-bound glutathione (GSS-X) were subjected to immunoblot analysis for PKM2. Representative blots of three independent experiments with consistent results are depicted.

The corrected legend appears below.

“Sfn does not affect Y105 phosphorylation but glutathionylation of PKM2 in M1 (LPS) macrophages. iBMDM were pretreated with DMSO or the indicated concentrations of Sfn for 30 min prior to stimulation with LPS for 6 h. **(A)** Total cell lysates were immunoblotted for p(Y105), total PKM2, and actin as a loading control. Representative blots of at least three independent experiments are depicted. Bar graphs represent compiled densitometric data. **(B)** Macrophages were pretreated with Sfn (10 µM) or the GSH donor glutathione-ethylester (GSEE) before LPS was added for another 6 h. Proteins were harvested under nonreducing conditions. Lysates (input) or immunoprecipitates obtained with an antibody specific for protein-bound glutathione (GSS-X) were subjected to immunoblot analysis for PKM2. Representative blots of three independent experiments with consistent results are depicted.”

The authors apologize for this error and state that this does not change the scientific conclusions of the article in any way. The original article has been updated.

In the published article, there was an error in **Figure 7**, panel B as published. The labeling of the lanes in the immunoblots was incorrect. The corrected **Figure 7** appears below.

The authors apologize for this error and state that this does not change the scientific conclusions of the article in any way. The original article has been updated.

