# Supplementary material for: Sulforaphane diminishes moonlighting of pyruvate kinase M2 and interleukin 1β expression in M1 (LPS) macrophages
Source: Front Immunol. 2022 Aug 2;13:935692. doi: 10.3389/fimmu.2022.935692 (PMC9380596; doi:10.3389/fimmu.2022.935692)
Supplement: Supplementary file 1 [file DataSheet_1.pdf]

SUPPLEMENTAL MATERIAL

Sulforaphane diminishes moonlighting of pyruvate kinase M2 and interleukin 1β expression in M1 (LPS) macrophages

Sheyda Bahiraii[1,2], Martin Brenner[1,2,3], Fangfang Yan[1,4], Wolfram Weckwerth[3,5] and Elke H Heiss[1*]

[1]Department of Pharmaceutical Sciences, University of Vienna, Vienna, Austria

[2] Vienna Doctoral School of Pharmaceutical, Nutritional and Sport Sciences (VDS PhaNuSpo), University of Vienna

[3]Vienna Metabolomics Center (VIME), University of Vienna, Vienna, Austria

[4]College of Food Science and Technology, Huazhong Agricultural University, Wuhan, China

[5]Molecular Systems Biology (MOSYS), Department of Functional and Evolutionary Ecology, University of Vienna, Vienna, Austria

Materials and Methods

Extra Antibodies

The anti-hexokinase1 (HK1) (#2024), anti-HK2 (#2867), anti-phosphofructokinase (PFK)2 (#13123) antibodies were from Cell Signaling Technology, the anti-PFK1 (#NBP-2 32170) was from Novus Biologicals and the anti-Nrf2 antibody from ProteinTech (#16396-1-AP). Those antibodies were used at dilutions of 1:1000.

Assessment of cell viability and biomass

Cells were seeded in 96-well plates and treated with 1-20 µM Sulforaphane and 100 nM Staurosporine as a positive control for 24 h. Cells were washed with PBS and incubated with 10 µg/mL Resazurin sodium salt (#R7017-Sigma Aldrich) in the serum-free DMEM medium containing 2% BSA for 4 h. Fluorescence at 580 nm excitation wavelength was measured with a microplate spectrophotometer (TECAN Sunrise™ Austria). Resazurin assay was followed by Crystal Violet staining to determine the number of attached cells. Briefly, cells were washed with PBS and stained with crystal violet solution for 5 min at room temperature. The

excessive dye was rinsed off with water and after drying at room temperature an EtOH/Citrate solution was added in order to solubilize crystal violet. The biomass was quantified by spectrophotometry at 595 nm with a TECAN Sunrise™.

Determination of cellular glucose uptake rates

Murine macrophages were seeded in 12-well plates. After treatment as indicated cells were equilibrated in standard Krebs Ringer Phosphate HEPES (KRPH) buffer containing 0.2% bovine serum albumin (BSA) for 20 minutes. The glucose uptake was initiated by addition of 2-DOG spiked with 2-deoxy-D-(1H3)-glucose (NEN Amersham, final concentrations 0.1 mM and 0.45 µCi/mL). After 15 min the reaction was stopped by three rapid washes with ice-cold PBS. The glucose uptake rate was determined by liquid scintillation counting (Perkin Elmer, Brunn am Gebirge, Austria) of cell lysates (lysis by 0.05 N NaOH in PBS), normalized to protein content assessed by the Rotiquant™ (Carl Roth, Karlsruhe, Germany) protein assay and uptake time (to obtain incorporated radioactivity per mg protein and minute) and corrected for the non-transporter-mediated glucose uptake (which is not inhibited by co-treatment with cytochalasin B (10 µM) during the uptake procedure).

LC/MS-based analysis of glutathione (oxidized and reduced)

$1.5x10^6$ (cells/ well) were seeded and the same sample preparation methods were performed as mentioned for GC-based analysis in the main manuscript. Samples were diluted in 50 µL of 0.1% formic acid in ddH2O, shortly vortexed and sonicated for 30 seconds. Afterwards, the solution was transferred to glass vials with inserts and sealed with crimp caps. Glutathione (reduced, 15494589, Fisher Scientific) and glutathione (oxidized, 10722895, Fisher Scientific) were measured as a quality control mix for identification.

The samples were separated on an Accucore™ Vanquish™ C-18+ UHPLC column (100·2.1 mm; 1.5 µm particle size) equipped with a guard column (Accucore™ Defender guards pk4, 10·2.1 mm, 2.5 µm particle size, both Thermo Fisher Scientific, Waltham, MA, United States). 5 µL of sample were injected with a flowrate of 0.250 ml/min. Mobile phase solvent A consisted of 0.1% FA in ddH2O and solvent B of MeOH containing 0.1%FA. A multistep gradient was used with the following steps: 0-1 min 0% B, 1-7 min linear increase to 25% B, 7-15 min linear increase to 99% B, 15-30 min stable at 99% B, 30.0 – 30.1 min linear decrease to 0% B, 30.1 – 40 min stable at 0% B.

Samples were measured on an Orbitrap Elite Instrument (Thermo Fisher Scientific, Waltham, MA, United States) with the following parameters: 50 – 2000 m/z in positive mode, Decamethylcyclopentasiloxane was used as lock mass with a m/z of 371.101230. For every MS1 fullscan, 5 data dependent MS2 scans (CID, 35

eV) were performed with the following mass list: cinnamic acid (149.05970, 35.0 eV), GSH (308.09108, 35.0 eV) and GSSG (613.15924, 35.0 eV). A dynamic exclusion list was generated with 500 entries for 60 seconds.

Generated files were converted into.mzml utilizing Proteowizard (version 3.0.21308, https://doi.org/10.1038/nbt.2377) and imported into MS-Dial (version 4.80, 10.1038/nmeth.3393). Area of the metabolites was normalized to the internal standard cinnamic acid and to the cell number from an experiment performed in parallel with 3 replicates. GSH/GSSG ratio was calculated using Excel (Microsoft Corporation, Redmond, WA, USA) and the figure generated utilizing Metaboanalyst.

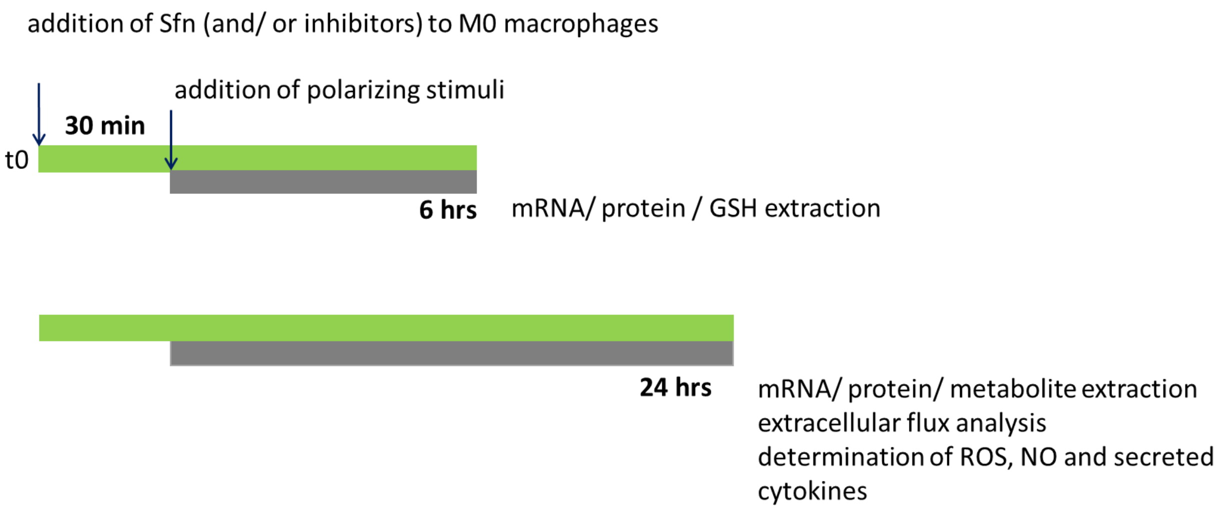

Supplemental Figure 1: Schematic diagram of the used experimental protocol

Cultivated M0 macrophages were treated with Sfn (+ inhibitors, if appropriate) at the indicated concentrations for 30 minutes before polarizing stimuli (LPS: 25 ng/mL; IL4: 20 ng/mL) were added for 6 or 24 hours. Workup for analysis of mRNA or protein expression, intracellular ROS, production of NO or metabolite levels followed the protocols given in the *Materials and Methods* section.

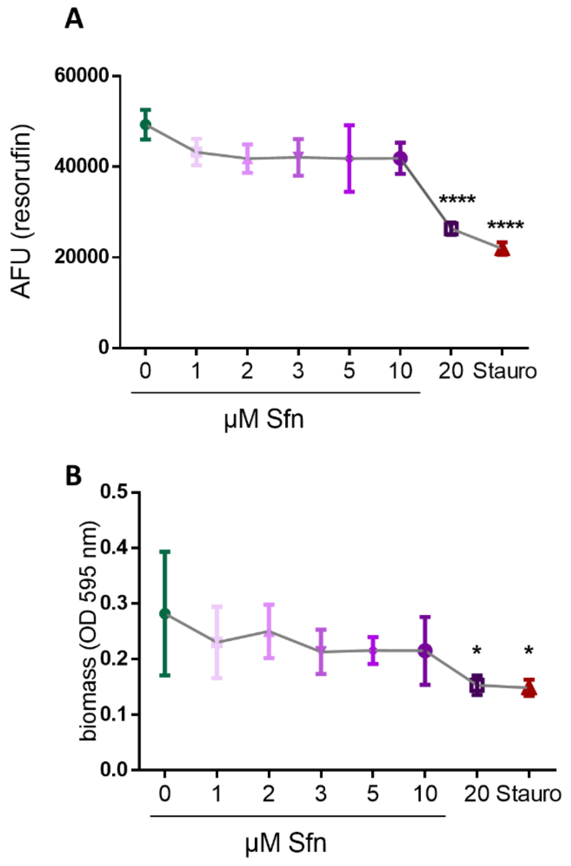

Supplemental Figure 2: Sfn does not impede cell viability of iBMDM in concentrations between 1 and 10 µM

iBMDM were treated with the indicated concentrations of Sfn or 100 nM staurosporine (stauro; positive control) for 24 h before cell viability was assessed by monitoring conversion of resazurin to resorufin and subsequent fluorescence measurement (A). Attached biomass was additionally stained by crystal violet (B).

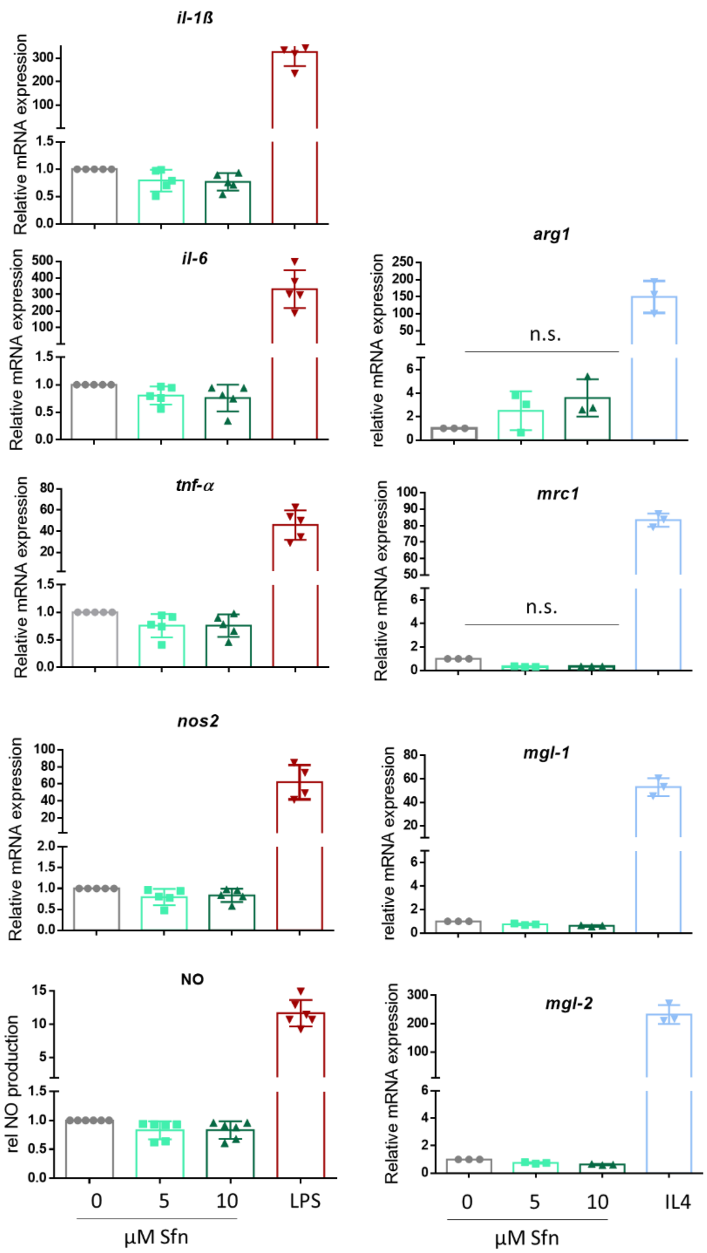

Supplemental Figure 3: Sfn does not influence expression of M1 and M2 markers in naïve M0 macrophages.

Naïve iBMDM were treated with the indicated concentrations of Sfn for 24 hours before expression of the indicated M1 and M2 markers was determined on the mRNA level by qPCR analysis (as described in detail in the main manuscript; n≥3, mean ± SD) or, as in the case of NO, the Griess assay was performed with cell culture supernatants. Macrophages stimulated with LPS (25 ng/mL) or IL4 (20 ng/mL) for 24 hours served as positive reference for M1 and M2 macrophages.

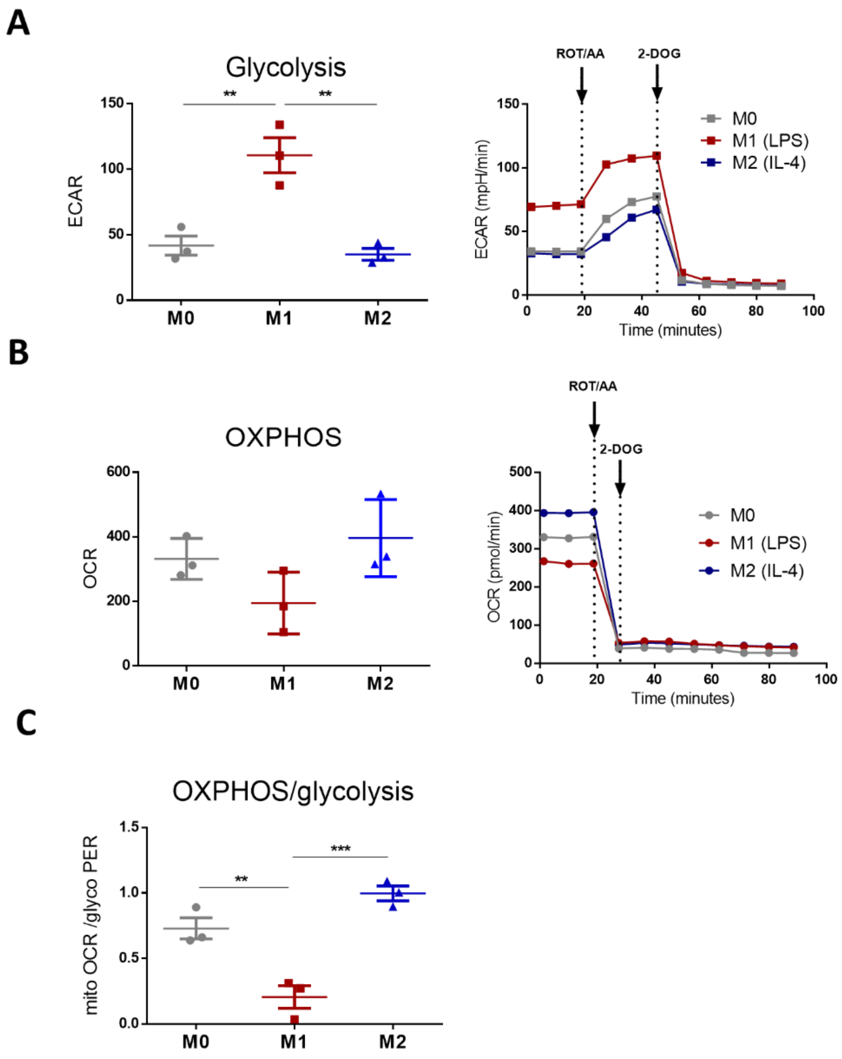

Supplemental Figure 4: Polarization of iBMDM by LPS and IL4 show the expected metabolic profiles of M0, M1 and M2 macrophages.

IBMDM macrophages were left untreated (M0), treated with LPS (25 ng/mL; M1) or IL4 (20 ng/mL, M2) for 24 h. Cells were then subjected to a glycolytic rate assay and extracellular flux analysis as described in detail in the *Methods* section. (A) depicts basal glycolytic activity (as evident in extracellular acidification rate (ECAR) in mpH/min), (B) OXPHOS activity (as evident oxygen consumption rate (OCR) in pmol/min) and (C) the ratio between basal mitoOCR/ glycoPER. Scatter dot blots (left) depict compiled data (basal ECAR and OCR) from three different biological replicates given as mean +/- SD. (** $p < 0.01$; *** $p < 0.001$, ANOVA, followed by multiple comparisons test). Graphs on the right show exemplary data from the performed glycolytic rate assays.

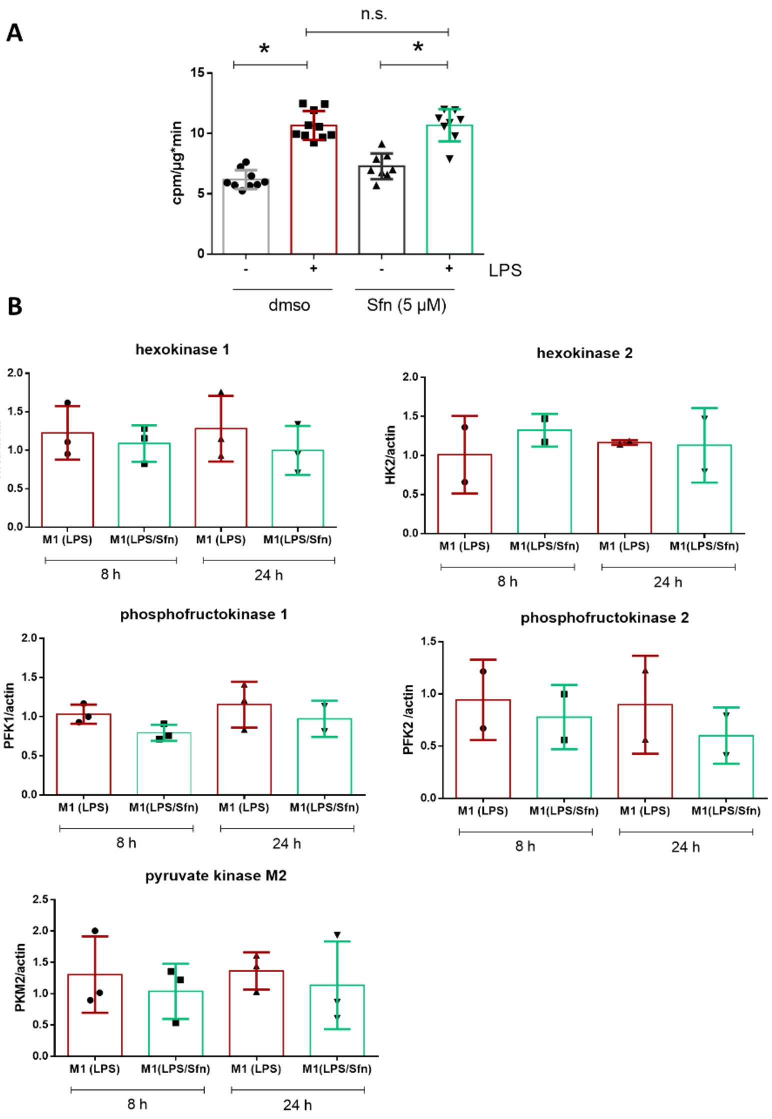

Supplemental Figure 5: Sfn does neither change glucose uptake into M1 (LPS) macrophages nor expression levels of key glycolytic enzymes

Murine macrophages (J774.1) were pretreated with DMSO or Sfn (5 µM) for 30 min before they were stimulated with LPS (100 ng/mL) for another 24 h. Then uptake rates of radiolabeled glucose (expressed as cpm/min*µg protein) was assessed as described in the methods section. Data are derived from ≥ 5 independent experiments and depicted as mean +/- SD, * p< 0.05, ANOVA, Tuckey´s post-test). (B) iBMDM were pretreated with DMSO (= M1 (LPS)) or Sfn (5 µM) (= M1 (LPS/Sfn)) for 30 minutes before LPS (25 ng/mL) was added for another 8 or 24 h. After protein extraction total cell lysates were subjected to immunoblot analyses for hexokinase 1, 2, Fructokinase 1, 2 or pyruvate kinase M2 and actin as loading control. Data (expressed as the ratio between densitometric signal of target and actin) derive from at least two independent experiments and are depicted as mean +/- SD.

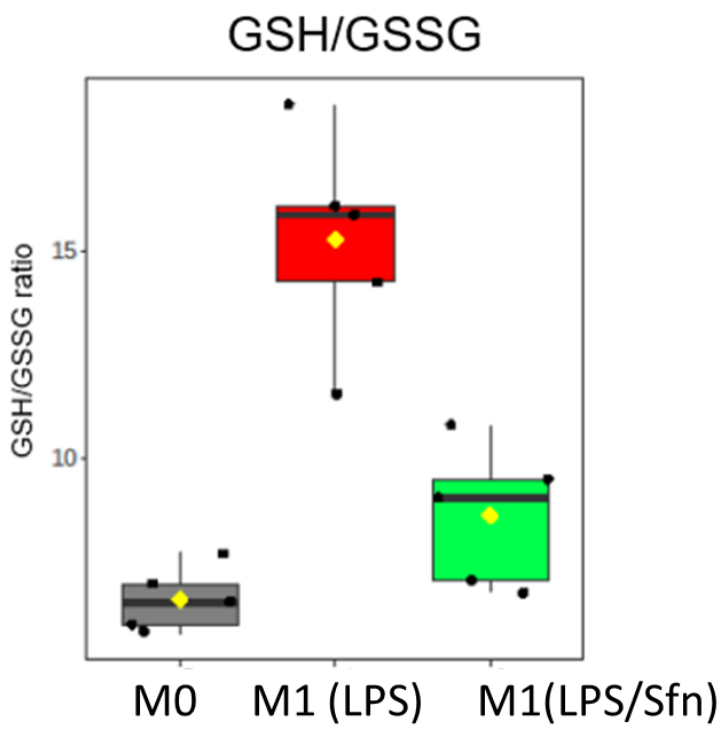

Supplemental Figure 6: Ratio of reduced (GSH) and oxidized glutathione (GSSG) in iBMDM

iBMDM were pretreated with Sfn (5 µM) for 30 minutes prior to stimulation with LPS (25 ng/mL) for 6 h and subsequent LC/MS-based analysis of GHSH and GSSG (described in detail in the methods section).

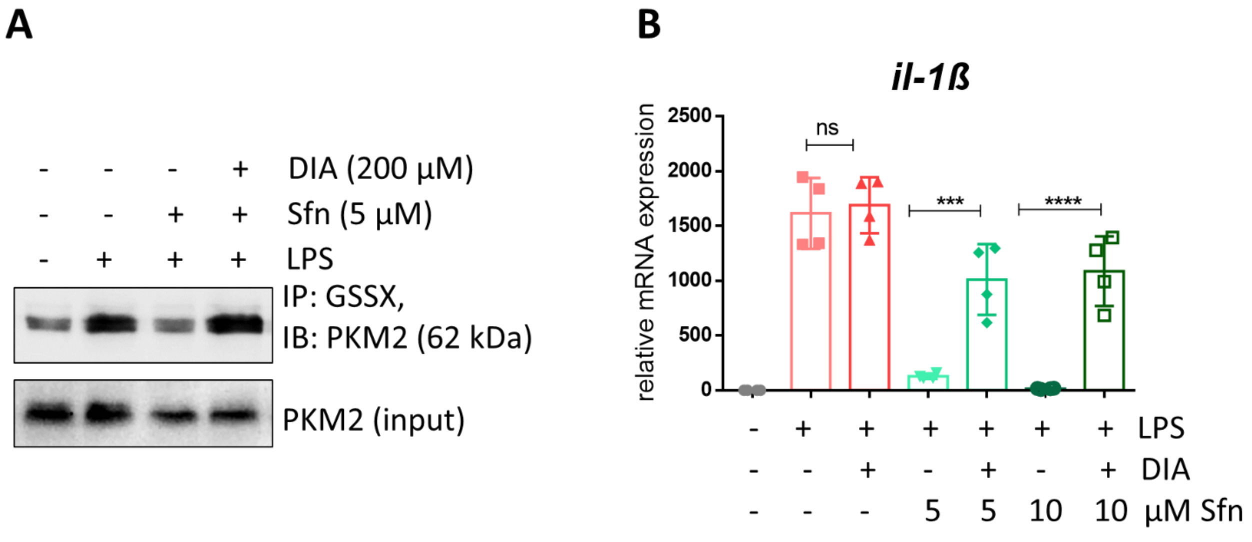

Supplemental Figure 7: Forced PKM2 gluthationylation by diamide blunts Sfn-mediated inhibition of IL1β expression

iBMDM were pretreated with diamide (DIA, 200 µM) and/or Sfn (5 or 10 µM) for 30 minutes prior to stimulation with LPS (25 ng/mL) for 6 h before (A) their lysates were subjected to immunoprecipitation with anti-DSSX antibody and then probed for PKM2. Aliquots of lysates prior to immunoprecipitation served as input control. Or, (B) RNA was isolated and analyzed by qPCR for *il1β* expression (*ppia* as reference) (n=3; mean +/- SD, ANOVA, followed by multiple comparisons test; *** $p < 0.001$; ****$p < 0.0001$).

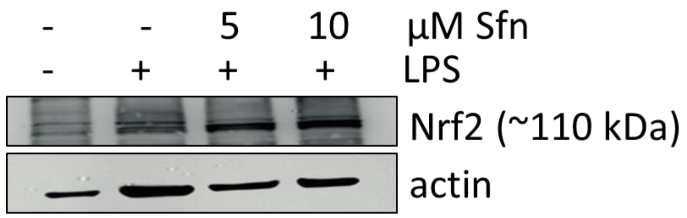

Supplemental Figure 8: Sfn leads to stabilization of Nrf2 in macrophages

iBMDM were pretreated with Sfn (5 and 10 µM) for 30 min prior to stimulation with LPS for 6 hrs. Total cell lysates were probed for Nrf2 and actin. One representative blot of three independent experiments with consistent results is depicted.
